# A predicted-loss based active learning approach for robust cancer pathology image analysis in the workplace

**DOI:** 10.1186/s12880-023-01170-8

**Published:** 2024-01-02

**Authors:** Mujin Kim, Willmer Rafell Quiñones Robles, Young Sin Ko, Bryan Wong, Sol Lee, Mun Yong Yi

**Affiliations:** 1 Graduate School of Data Science, Department of Industrial and Systems Engineering, Korea Advanced Institute of Science and Technology, Daejeon, South Korea; 2 Pathology Center, Seegene Medical Foundation, Seoul, South Korea

**Keywords:** Active learning strategy, Noisy data, Cancer pathology images, Convolutional neural networks, Deep learning, Histopathology image analysis, Predicted loss

## Abstract

**Background:**

Convolutional neural network-based image processing research is actively being conducted for pathology image analysis. As a convolutional neural network model requires a large amount of image data for training, active learning (AL) has been developed to produce efficient learning with a small amount of training data. However, existing studies have not specifically considered the characteristics of pathological data collected from the workplace. For various reasons, noisy patches can be selected instead of clean patches during AL, thereby reducing its efficiency. This study proposes an effective AL method for cancer pathology that works robustly on noisy datasets.

**Methods:**

Our proposed method to develop a robust AL approach for noisy histopathology datasets consists of the following three steps: 1) training a loss prediction module, 2) collecting predicted loss values, and 3) sampling data for labeling. This proposed method calculates the amount of information in unlabeled data as predicted loss values and removes noisy data based on predicted loss values to reduce the rate at which noisy data are selected from the unlabeled dataset. We identified a suitable threshold for optimizing the efficiency of AL through sensitivity analysis.

**Results:**

We compared the results obtained with the identified threshold with those of existing representative AL methods. In the final iteration, the proposed method achieved a performance of 91.7% on the noisy dataset and 92.4% on the clean dataset, resulting in a performance reduction of less than 1%. Concomitantly, the noise selection ratio averaged only 2.93% on each iteration.

**Conclusions:**

The proposed AL method showed robust performance on datasets containing noisy data by avoiding data selection in predictive loss intervals where noisy data are likely to be distributed. The proposed method contributes to medical image analysis by screening data and producing a robust and effective classification model tailored for cancer pathology image processing in the workplace.

## Background

Cancer is a major cause of death worldwide, characterized by high heterogeneity and significant barriers to extending human life expectancy [1, 2]. According to a World Health Organization survey, cancer is the leading or second leading cause of death [3]. The current standard for diagnosing cancer involves pathologists reviewing glass slides with stained suspicious tissue under a high-power microscope [4]. However, with an annual increase in cancer cases and a relatively scarce number of pathologists, the workload of pathologists has increased, leading to approximately 3–9% of human errors in anatomical pathology [5, 6]. To reduce workload, there has been active research on deep learning (DL)-based models that analyze digitized whole-slide images (WSIs) [7–9].

To develop a DL-based model that analyzes WSIs, it is common to use “patch images,” which are relatively small images generated from WSIs [10–12]. WSIs are large digital images of actual slides created by a scanner, typically consisting of many gigapixels; up to 50,000 × 50,000 pixels. If we apply convolutional neural networks (CNNs), which are a representative DL architecture for image processing, directly to WSI classification, there can be two significant drawbacks. First, down-sampling can result in the loss of detailed information, and second, CNNs can only learn some of the distinctive patterns that appear in multiple WSIs [13]. Therefore, it is advantageous to train a CNN with high-resolution patch images and predict a label for a WSI on the basis of patch-level information.

**Fig. 1:**
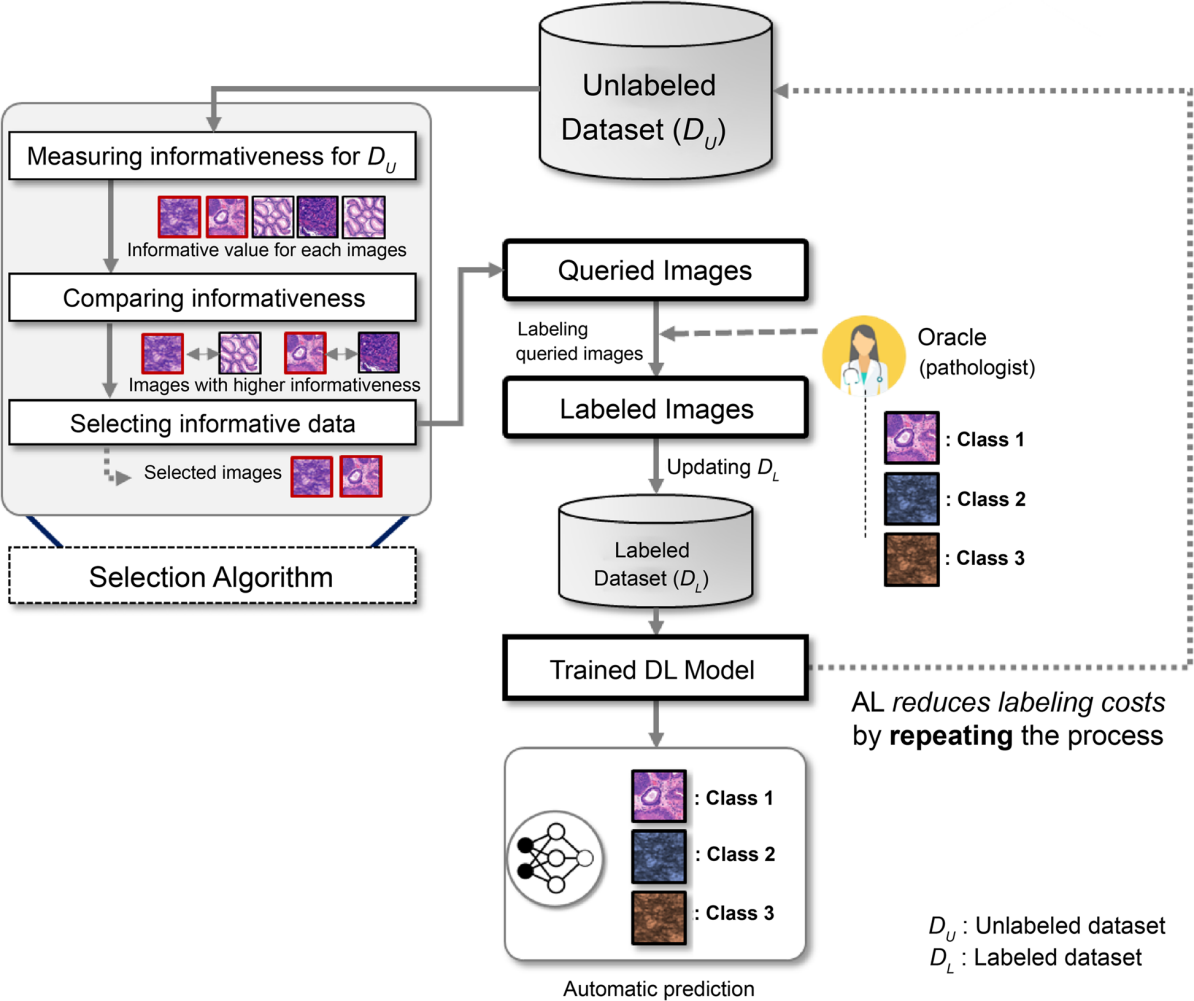
Concept diagram of typical active learning (AL) framework. DL: deep learning

These high-performing CNN models require large amounts of labeled data [14]. The main challenge in building a high-quality dataset for CNN training is the labor-intensive and time-consuming process of labeling medical images by expensive medical experts [15]. Recently, various methods, such as semi-supervision, transfer learning, and multi-instance learning, have been studied to overcome this labeling issue in medical image analysis [16]. Active learning (AL) is one approach that focuses on acquiring labels for the most informative data in an efficient manner, exploring how to efficiently acquire “real” labels. Unlike other methods, AL allows the DL model to actively select and preferentially label the most informative data obtained from medical experts, to optimize the trade-off between labeling efforts and model performances [17]. The AL framework typically consists of a method for measuring the informativeness of each unlabeled data point, as shown in Fig. 1.

First, as shown in Fig. 1, the model selects informative data using a selection algorithm from an unlabeled dataset and requests an oracle (i.e., medical expert) to label the queried images. Second, these labeled data are used to train the DL model. By repeating this process, the size of the labeled dataset gradually increases. Through this framework, a high-performance DL model can be trained at a low labeling cost.

In the context of AL, a model is initially trained with a small amount of labeled data (typically an arbitrary number selected by a researcher such as 40 [18], or 500 [15]) and then repeatedly selects data to request labeling from an oracle using an acquisition function (query), often based on the model’s uncertainty information [19]. Various AL strategies have been proposed for medical image analysis to reduce labeling costs. For instance, several studies have explored uncertainty in nuclear segmentation within histopathological images, with some focusing on utilizing the posterior probability of the output to compare model performance for breast and pancreatic cancer [20]. Another study introduced methods for measuring uncertainty using Bayesian CNNs specifically for skin cancer [19], while a more recent study proposed an uncertainty measurement approach that comprehensively considers both entropy and high confidence scores in the context of breast cancer [21].

Recently, in the field of digital pathology, there have been studies that combine methods for removing false-labeled patch images with uncertainty-based AL strategies [22], or that consider both uncertainty and representation in patch-based analysis [15]. However, the use of an AL strategy based on uncertainty can be challenging when dealing with noisy real-world industrial data, as most DL studies use clean or minimally noisy (dirty) publicly available datasets, potentially worsening performance when noisy samples are queried [23]. Noisy images can be generated in the workplace due to various issues, such as out-of-focus scanning, missing tissue, air bubbles, poor staining, poor sectioning, tissue artifacts, tissue folding, or poor dehydration [24, 25], leading to poor quality patch images. Therefore, there is a dire need to develop AL strategies that are suitable for noisy real-world industrial datasets.

In this study, we proposed a novel AL strategy for analyzing histopathological images that minimize the selection of noisy data when querying data from an unlabeled set. The purpose of this study was to construct an AL strategy that can continuously improve the performance of histopathological patch classification models in situations where noisy data are included in the dataset. The proposed AL strategy was based on learning loss (LL) [26] and used a modified version of LossDiff [27] in the sampling stage of AL.

## Methods

**Fig. 2:**
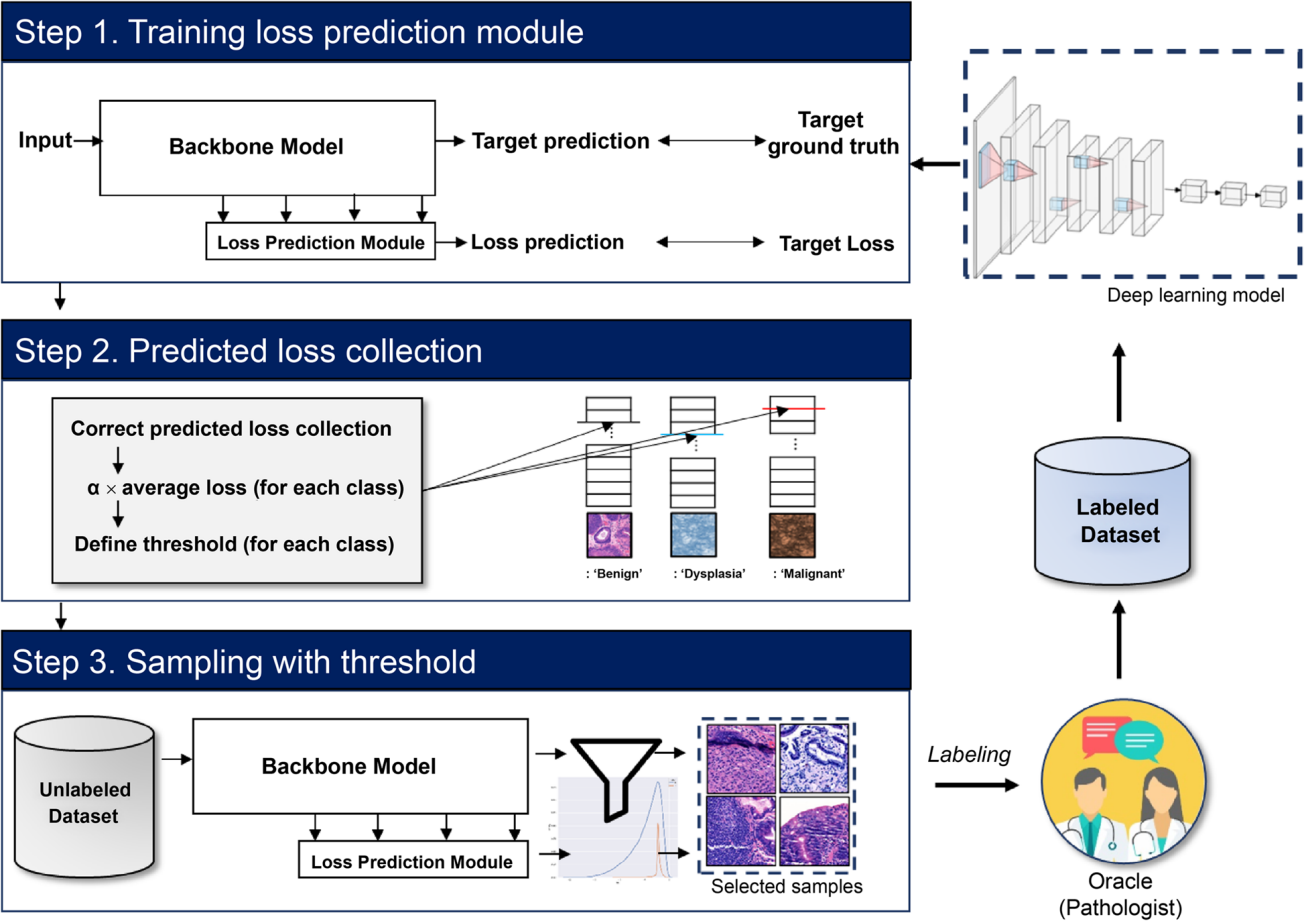
The overall process of the proposed method

The primary goal of this research was to develop a robust AL approach for noisy histopathology datasets. The method consisted of three steps: 1) training a loss prediction module (LPM), 2) collecting predicted loss values, and 3) sampling data for labeling (Fig. 2).

As shown in Fig. 2, in the training loss prediction module (LPM) step, the backbone deep learning model and the LPM are trained. In the predicted loss values collection step, the predicted loss value of the correctly predicted data is collected to calculate the appropriate threshold values. Next, in the sampling step, the model calculates the prediction loss to select informative data from ${D}_{U}$, which is filtered by the threshold value calculated in the training step, and then the high uncertainty data is sampled.

### Training LPM step

**Fig. 3:**
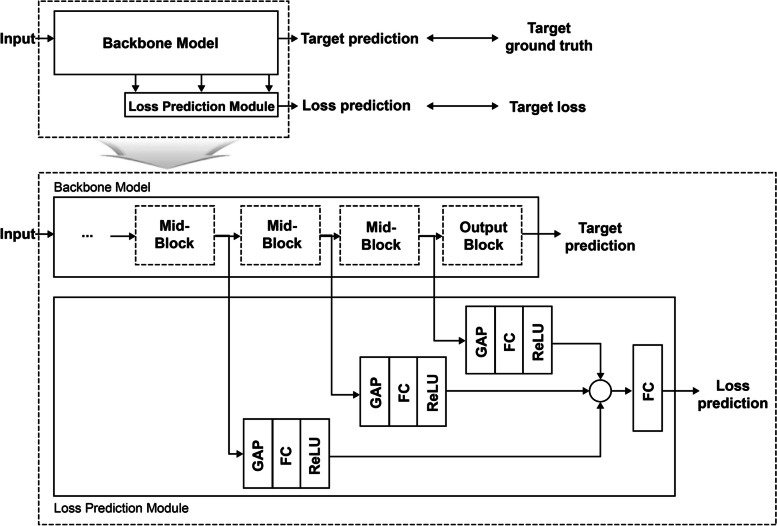
A conceptual diagram of the Loss Prediction Module (LPM). GAP: global average pooling; FC: full connected layer; ReLU: Rectified Linear Unit

The first step of the proposed AL method was handled by the LPM. The LPM is attached to a deep network and trained with the backbone to predict the loss of input data. Therefore, LL is expected to be widely useful, as LPMs can be attached to any kind of DL networks. In this study we used Visual Geometry Group-16 (VGG-16, VGG Group, Oxford, UK) [28] as the backbone architecture, without dropout. Figure 3 depicts a conceptual diagram of the LPM, in which the second diagram depicts an expanded view of the first one.

To train the LPM simultaneously with the backbone model, we calculated the total loss value by summing the target model loss originating from the backbone and the loss of the LPM. The overall loss function is represented by Eq. (1).

1 $${L}_{target}\left(\widehat{y},\mathrm{ y}\right)+\uplambda \cdot {L}_{loss}(\widehat{l}, l)$$

In addition, it is recommended to use the margin-ranking loss function to train the LPM for better performance [26]. The margin-ranking loss is a loss function that pairs samples within a batch to compare ranks. If the size of the mini-batch is *B*, we can create a *B/2* data pair and train the LPM by considering the differences between the loss prediction pairs. Therefore, the loss function for the LPM was defined as follows:

2 $$\begin{array}{l}{L}_{loss}\left(\widehat{{l}^{p}}, {l}^{p}\right)={\text{max}}\left(0,-\mathbb{1}\left({l}_{i},{l}_{j}\right)\cdot \left({\widehat{l}}_{i}-{\widehat{l}}_{j}\right)+\varepsilon \right)\\ {\text{s}}.{\text{t}}.\mathbb{1}\left({l}_{i},{l}_{j}\right)=\left\{\begin{array}{c} +1, if {\widehat{l}}_{i}>{\widehat{l}}_{j} \\ -1, otherwise\end{array}\right.\end{array}$$

$\varepsilon$ is the predefined positive margin and *p* is the pair of *i, j*. For example, if ${l}_{i}$ is larger than ${l}_{j}$ and ${\widehat{l}}_{i}$ is greater than ${\widehat{l}}_{j}$+$\varepsilon$, the loss value is 0. By using this loss function, the LPM can learn to distinguish between informative and non-informative data and predict the loss value more accurately. The following Algorithm 1 represents the algorithm for training the LPM

**Figure:**
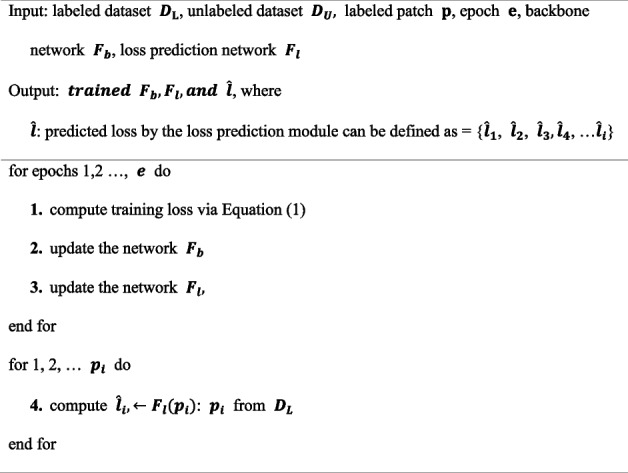
**Algorithm 1.** Loss prediction module

### Predicted loss collection step

One of the essential components of the proposed AL method is the predicted loss collection module (PLCM). The PLCM generates an appropriate threshold for each class based on the predicted loss value. Specifically, the PLCM observes the predicted loss (${b}_{l}$) based on the baseline labeled dataset (${D}_{L}$). At this point, the loss results for instances that have been correctly classified as $y=\widehat{y}$ for each patch type (class) $t$, where $y$ is the ground truth, $\widehat{y}$ is the model prediction, and $t \in \left\{D, M, N\right\}$, where D, M, N stands for a different disease class, respectively (the definitions of these disease classes are provided in *Dataset construction* Section), were recorded. For a batch $b$ of $m$ instances, the loss for correctly classified instances can be defined as ${b}_{cl}=\left\{{\widehat{l}}_{c1}, {\widehat{l}}_{c2}, {\widehat{l}}_{c3}\dots {\widehat{l}}_{cn}\right\}$, where ${\widehat{l}}_{cn}$ denotes the predicted loss $l$ of $n$ correctly classified instances $c$. In addition, the loss for correctly classified instances and each patch type $t$ within a batch were recorded and the average loss was obtained using the following Eq. (3):

3 $${b}_{cl(avg)}=\left(\frac{{\sum }_{i=1}^{n}{l}_{ci}}{n}\right)$$

where *n* is the total number of correctly classified instance *c*. Furthermore, while ${b}_{cl(avg)}$ was obtained from each batch, the final average value of ${b}_{cl\left(avg\right)final t}$ was obtained by collecting all ${b}_{cl(avg)}$ from the last z epochs, as shown in Eq. (4), where *k* is the number of batches in each epoch.

4 $${b}_{cl\left(avg\right)final\,t} \leftarrow \left[{\left(\frac{{\sum }_{j=1}^{k}{b}_{cl\left(avg\right)}j}{k}\right)}_{1}+{\left(\frac{{\sum }_{j=1}^{k}{b}_{cl\left(avg\right)}j}{k}\right)}_{2}+\dots +{\left(\frac{{\sum }_{j=1}^{k}{b}_{cl\left(avg\right)}j}{k}\right)}_{z}\right]\frac{1}{z}$$

In addition, it should be noted that ${b}_{cl\left(avg\right)final t}$ has a different value depending on the patch type (i.e., D, M, N), thus if the number of the patch type is multiple *t, *${b}_{cl\left(avg\right)final}$*=*{${b}_{cl\left(avg\right)final 1} , {b}_{cl\left(avg\right)final 2}, \dots , {b}_{cl\left(avg\right)final t}$}. Finally, to avoid filtering out difficult cases, $\alpha$ was used as a hyperparameter and was multiplied to ${b}_{cl\left(avg\right)final t}$ for generating a threshold that could be separately set for each patch type *t*, as shown in Eq. (5).

5 $${threshold}_{t}= {a}_{t}*{b}_{cl\left(avg\right)\mathrm{final\, }t}$$

The following Algorithm 2 represents the algorithm for training predicted loss collection.

**Figure:**
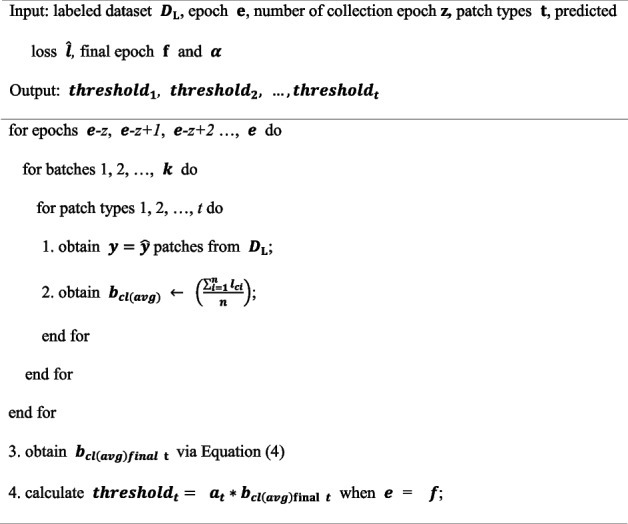
**Algorithm 2.** Predicted loss collection

### Sampling step

In the last step, the sampling module seeks to select informative samples, which is done by predicting the loss value in the LPM for all of the data contained in the ${D}_{U}$. For a clean dataset, data with the highest predicted loss can be assumed to be the most informative. However, for a noisy dataset, this assumption may not hold. Therefore, we sought to avoid selecting noisy data by removing those data with excessively high loss values from the selection candidates, based on the threshold calculated by the PLCM. Specifically, data with $\widehat{l}$ $\ge$ ${threshold}_{t}$ for the predicated loss, were excluded from the selection candidates [27]. In other words, our sampling module selected the top–*k* loss value data based on loss value among the data that satisfy “$\widehat{l}$* < *${threshold}_{t},$*”* where *k* is the number of samples selected for a particular class. Because each threshold and data selection were conducted based on the predicted patch type by the backbone model, the threshold for the predicted class was used to exclude noisy data, and the same amount of data was sampled for each prediction class. Finally, the sampled data were labeled by an oracle and utilized for model training. After the sampling step, we defined ${D}_{U i+1}$ and ${D}_{L i+1}$, which were the datasets for the next iteration, as follows: ${D}_{U i+1}$= ${D}_{U i}$ - ${p}_{l}$, ${D}_{L i+1}$= ${D}_{L i}$+ ${p}_{l}$, where ${p}_{l}$ denotes the patches labeled by the oracle and can be defined as = {${p}_{l1}$, ${p}_{l2}$, ${p}_{l3}$, …${p}_{ln}$}). A set of patches that maximize the sum of loss values when put into the loss prediction network can be defined as selected patches, following Equation (6), in which ${F}_{l}$ denotes loss prediction network and ${p}_{s}$ denotes selected (or sampled) patches. The following algorithm 3 represents the algorithm about the sampling for labeling.

6 $${{\text{argmax}}}_{S\subseteq {D}_{U},|S|\le k}\sum_{s}{F}_{l}({p}_{s})$$

**Figure:**
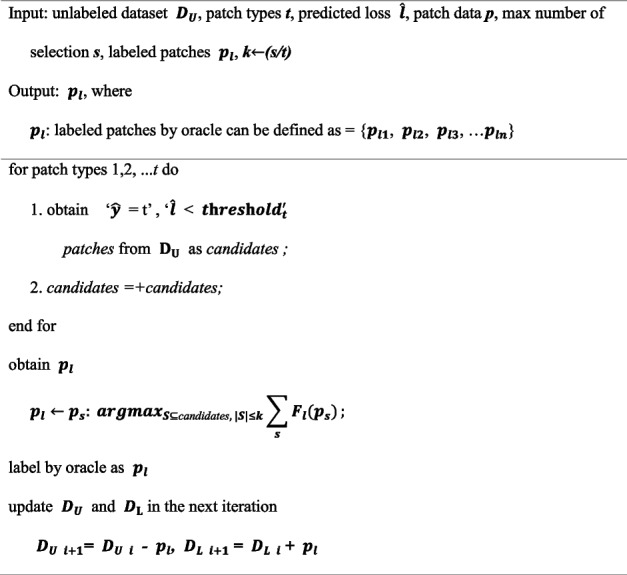
**Algorithm 3.** Sampling for labeling

### Experiment

#### Dataset construction

We constructed a large histopathology dataset extracted from stomach WSIs obtained from Seegene Medical Foundation, one of the largest diagnostic and pathology institutions in South Korea. These slides were stained with hematoxylin and eosin and scanned using a scanner (Pannoramic Flash250 III, 3DHISTECH, Budapest, Hungary) at 200× magnification. The data were collected by the medical foundation and their use for research was approved by the Institutional Review Board (SMF-IRB-2020-007) of Seegene Medical Foundation, as well as by the Institutional Review Board (KAIST-IRB-22-334, KH2020-116) of the Korea Advanced Institute of Science and Technology, the university that collaborated with the medical foundation. The medical foundation’s designated collection centers provided informed consent to use their tissue samples for clinical purposes. All experiments were performed in accordance with relevant guidelines and regulations provided by the two review boards. All patient records were completely anonymized, and all images were stored and analyzed only on the organization’s server.

To train the model, two types of datasets are needed: the unlabeled dataset (${D}_{U}$) and the labeled dataset ${(D}_{L})$. The unlabeled dataset is a dataset containing all candidate patches that require labeling. Patches included in the unlabeled dataset cannot be used for training a model because they do not have labels (i.e., correct answers for classification). Therefore, in the process of AL, the model selects some patches with the highest amount of information from the unlabeled dataset and asks an oracle for a label. On the contrary, the labeled dataset is a dataset that includes labels assigned by an oracle. In order to build a labeled dataset, we randomly selected 1,000 patches and used them in the first iteration in all experiments. However, in subsequent iterations, the set of patches that made up the labeled dataset were changed as the amount of information of the patches changed while performing the AL experiment. Accordingly, the patches constituting the unlabeled dataset continuously changed.

**Fig. 4:**
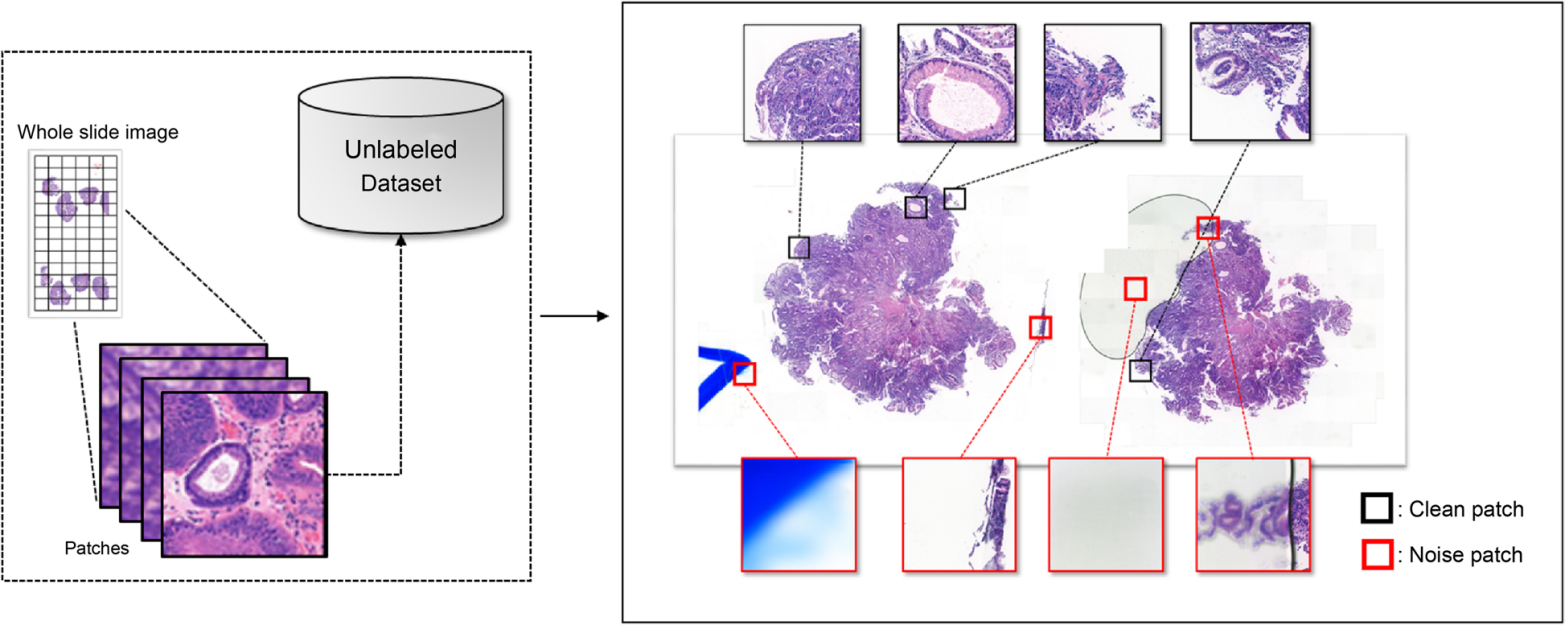
Patch image generation process. Generation using clean (black boxes) and noise (red boxes) patch images from a field pathology slide.

To construct the unlabeled dataset, we collected 600 WSIs from different patients, each of which was then converted into a number of 256 × 256-pixel-sized patches, resulting in a total of 118,531 patches. The overall process is depicted in the left box of Fig. 4. However, as mentioned earlier, poor-quality images can be generated due to scanning or slide quality issues, resulting in the generation of noisy (unclean) samples of patch images, as shown in the right box of Fig. 4. Out of the total patches, 6,920 were classified as noisy. In sum, the unlabeled dataset consisted of 111,611 clean patches and 6,920 noisy patches.

For this study’s experiment, patch images used for training were restricted to three classes: 1) benign, 2) dysplasia, and 3) malignant. “Benign” refers to a diagnosis of non-neoplastic benign gastric mucosal lesions, including gastritis and polyps. “Dysplasia” includes suspicious for (s/f) and suspicious of (s/o) tubular adenoma with dysplasia of any grade (s/f, s/o); while ‘Malignant’ covers malignant neoplasms, including adenocarcinoma, (s/f, s/o) adenocarcinoma, (s/f, s/o) high-grade lymphoma, and any other (s/f, s/o) carcinoma or malignant neoplasm. Thus, within the AL process, the model selected data from the ${D}_{U}$, which could be classified into three classes or noisy images by an oracle. In addition, the test dataset for evaluating the trained model’s performance with ${D}_{L}$ was constructed from images generated from 150 WSIs (50 per class), and a total of 30,523 patch images (11,753 Benign, 8,281 Dysplasia, and 10,489 Malignant) (Table 1).

**Table 1:** Summary of dataset construction

	Slide		Patch	
${D}_{U}$	Total	600	Clean	111,611
			Noisy	6,920
			Total	118,531
${D}_{L}$	-		1^st^ iteration	1,000
			Available cost, *k*	1,000
			Final iteration	10,000
Test	Benign	50	Benign	11,753
	Dysplasia	50	Dysplasia	8,281
	Malignant	50	Malignant	10,489
	Total	150	Total	30,523

Table 1 presents the dataset configuration, highlighting the ${D}_{U}$ inclusion of 118,531 patch images sourced from 600 WSIs. Among them, certain images lacked sufficient meaningful information for classification into benign, dysplasia, or malignant categories, resulting in noisy patches labels. There was a total of 6,920 noisy patches, accounting for approximately 5.8% of all patches. As the experiment advanced, ${D}_{L}$ systematically increased the patch count, reaching a maximum of 10,000 in the final iteration. Each iteration involved the selection of 1,000 samples. Additionally, test data, collected independently from ${D}_{U}$, were used to evaluate the model’s performance.

**Fig. 5:**
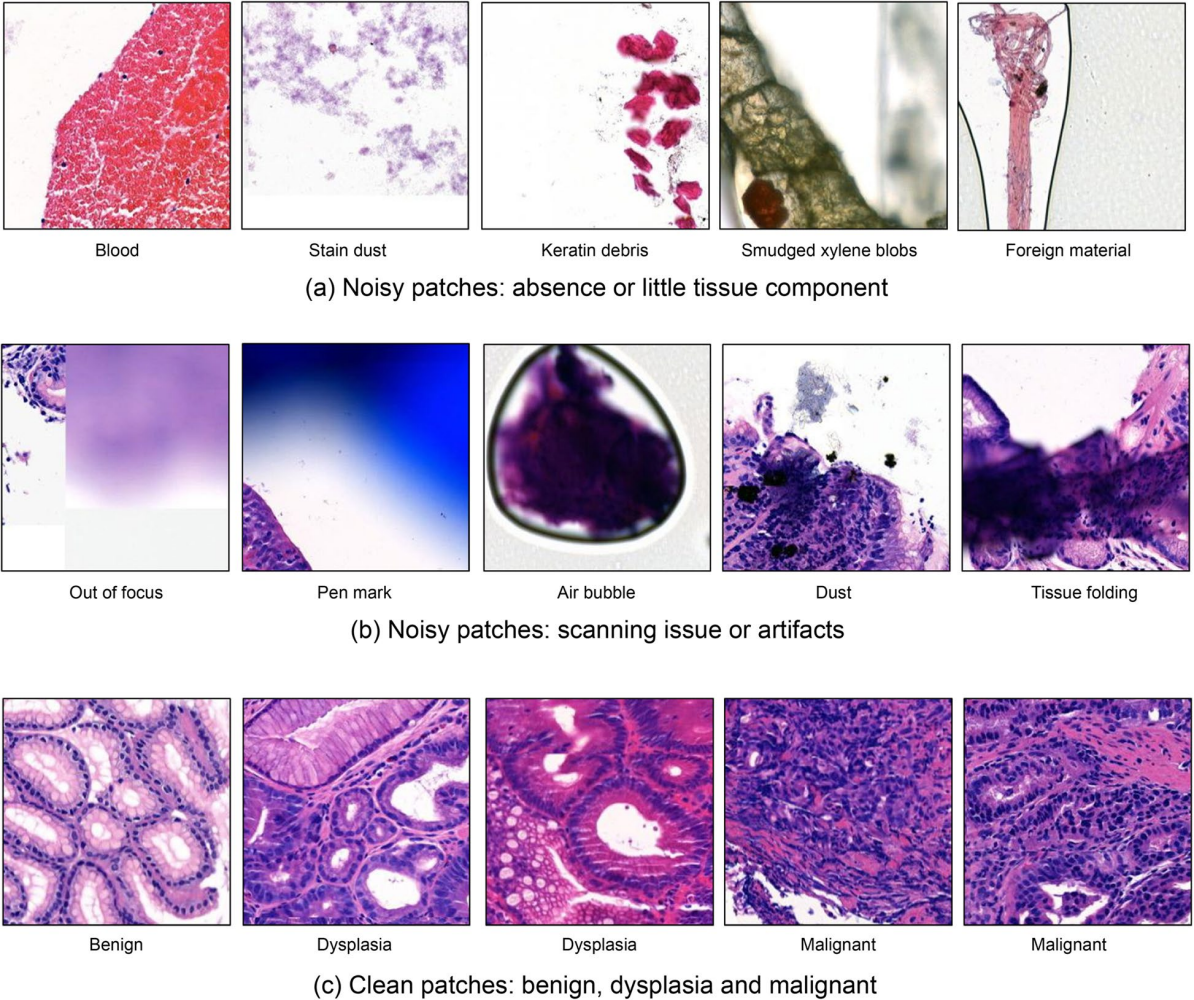
Examples of noisy and clean patches (**a**) noisy patches that occur during the imaging process, involving substances or objects that are irrelevant to the classification, or of no interest; **b** noisy patches where meaningful tissue imaging is hindered by scanner errors or the presence of other substances; **c** clean patches that contain sufficient information for an accurate classification by an oracle

Figure 5 shows examples of noisy and clean patches in ${D}_{U}$. Figure 5(a) shows patches with no or little tissue components. During the scanning process, typical examples include the presence of blood and stain dust. In some cases, foreign substances located outside of the tissue were inadvertently captured by the scanner and transformed into patches. Figure 5(b) specifically shows these patches, which presented difficulties in tissue capturing due to scanner errors or specific artifacts. Scanner focus-out is a frequently encountered error that can impede tissue capturing, and the presence of dust or air bubbles can also interfere with tissue capturing. In addition, there are patches that pose challenges in tissue classification due to folding that occurs during the slide creation process. In contrast, unlike the aforementioned noisy patches, clean patches contain sufficient tissue shape and information. Figure 5(c) displays these clean patches alongside their corresponding labels.

### Implementation details

The proposed method was implemented in Python (Wilmington, DA, USA), using the PyTorch library on a server equipped with two NVIDIA RTX 3080 TI GPUs (Nvidia Corporation, Santa Clara, CA, USA). The goal of this study was to develop a robust AL strategy for industrial applications using noisy data. In most AL studies, the efficacy of each AL method is typically assessed by observing the model's performance changes while varying data selection. Thus, tracking model performance changes associated with a chosen AL method is crucial. Because VGG16 possesses a relatively simpler structure than more advanced deep learning networks, it allowed us to easily track model performance. Moreover, instead of pretrained models, most AL research employs a learning method referred to as training from scratch, which involves training a neural network from the beginning. For these reasons, we chose VGG16 as our backbone model, training it from scratch. This CNN model was trained with the Stochastic Gradient Descent optimizer, and we set the batch size to 32, epoch size to 50, and learning rate to 0.001. Additionally, the backbone model used cross-entropy as a loss function, and the loss function of the LPM used the same margin-ranking loss as specified in Eq. (2).

Furthermore, we implemented 10 iterations of experiments to examine the effectiveness of the AL method. The “available cost” *k*, which is the number of data selected for labeling by the AL method, was set to the value of 1,000. By repeating this 10 times, a total of 10,000 patch images were selected in the last iteration. If noisy patches were selected during the AL process, they were excluded from the AL cycle because they could not be labeled. That is, ${p}_{n}$ is a set of noisy patches among the selected patches by the model, and ${p}_{l}$ is a set of label-able patches. In each iteration, two sets ${p}_{n}$ and ${p}_{l}$ were excluded from ${D}_{U}$, and ${p}_{l}$ was added to ${D}_{L}$. Therefore, it can be represented as ${D}_{U i+1}$ = ${D}_{U i}$ - ${p}_{n}$ - ${p}_{l}$,${D}_{L i+1}$ = ${D}_{L i}$ + ${p}_{l}$ in the next iteration (i+1). In this experiment, because the model selects 1,000 patches in each iteration, the sum of the elements of the two sets ${p}_{n}$ and ${p}_{l}$ in each iteration was 1,000 (i.e., |${p}_{n}|$ + |${p}_{l}|$ = 1,000).

### Comparison methods

To evaluate the effectiveness of the proposed method, we compared its performance by applying the proposed method and six different AL methods to the same backbone model. They were least confidence (LC), entropy, Bayesian AL by disagreement (BALD), LL, core-set, and random sampling (RS).

LC [29] queries the most uncertain examples with the lowest softmax confidence while predicting their labels. This method assumes that the model *n*-classes output nodes are denoted by $\overrightarrow{z}$ and each output node is denoted by ${z}_{j}$. Thus, *j* $\in$[1, *n*-classes]. Then, for an output node ${z}_{i}$ from the model, the corresponding softmax is

7 $$\upsigma \left({z}_{i}\right)=\frac{{e}^{{z}_{i}}}{\sum_{j}{e}^{{z}_{j}}}$$

The softmax can then use the selected number of data points to select the label for which the model has the lowest confidence, as follows:

8 $${{\text{argmin}}}_{S\subseteq {D}_{U},|S|\le k}\sum\nolimits_{S}({argmax}_{j}(\sigma (\overrightarrow{z})))$$

Entropy [30]**-**based AL computes the entropy from a softmax output vector. It is one of the basic AL methods that selects images for which the model is most uncertain. To quantify the uncertainty, entropy is used, and thus, images with maximum entropy are selected. While assuming the model has *n*-class output nodes and each output node is denoted by ${z}_{j}$ ( *j* $\in$[1, *n*-classes]), the entropy can then be calculated as:

9 $${\text{Entropy}}= - \sum_{j}\upsigma \left({z}_{j}\right)*{\text{log}}\left(\upsigma \left({z}_{j}\right)\right).$$

This algorithm selects *k* number of data points for label with the highest entropy.

BALD [19] is an AL method that operates under a Bayesian setting and selects data that maximizes the mutual information between the predicted labels and model parameters. To implement BALD, dropout layers are added to the DL model so that it can be performed in a non-Bayesian setting. Stochastic forward passes are then performed through the dropout, and the difference in prediction entropy (mutual information) is measured. Finally, the data with the highest mutual information is selected for labeling.

LL [26] queries examples with the highest predicted loss by jointly learning an LPM. Unlike other existing uncertainty-based AL methods that rely on additional predictive loss information from the model, LL uses a dedicated module to predict the loss values and selects data based on these predictions.

Core-set [31] is the most popular representativeness-based AL method, which places the data in a feature space and then selects the data that contain the most diverse samples as informative samples. Among the core-set-based methods, we used the *k*-greedy method. This method aims to select *k* data points that minimize the radius of the subgraph when placing data in the feature space and repeatedly selecting the furthest data point from one randomly selected data point to produce *k* subgraphs covering all data points. As a result, the core-set is a method for selecting the most divergent *k*-examples with the highest coverage in a representative space.

Finally, the RS method is a non-AL approach that randomly selects data without considering their uncertainty or representativeness.

## Results

### Predicted loss value analysis

**Fig. 6:**
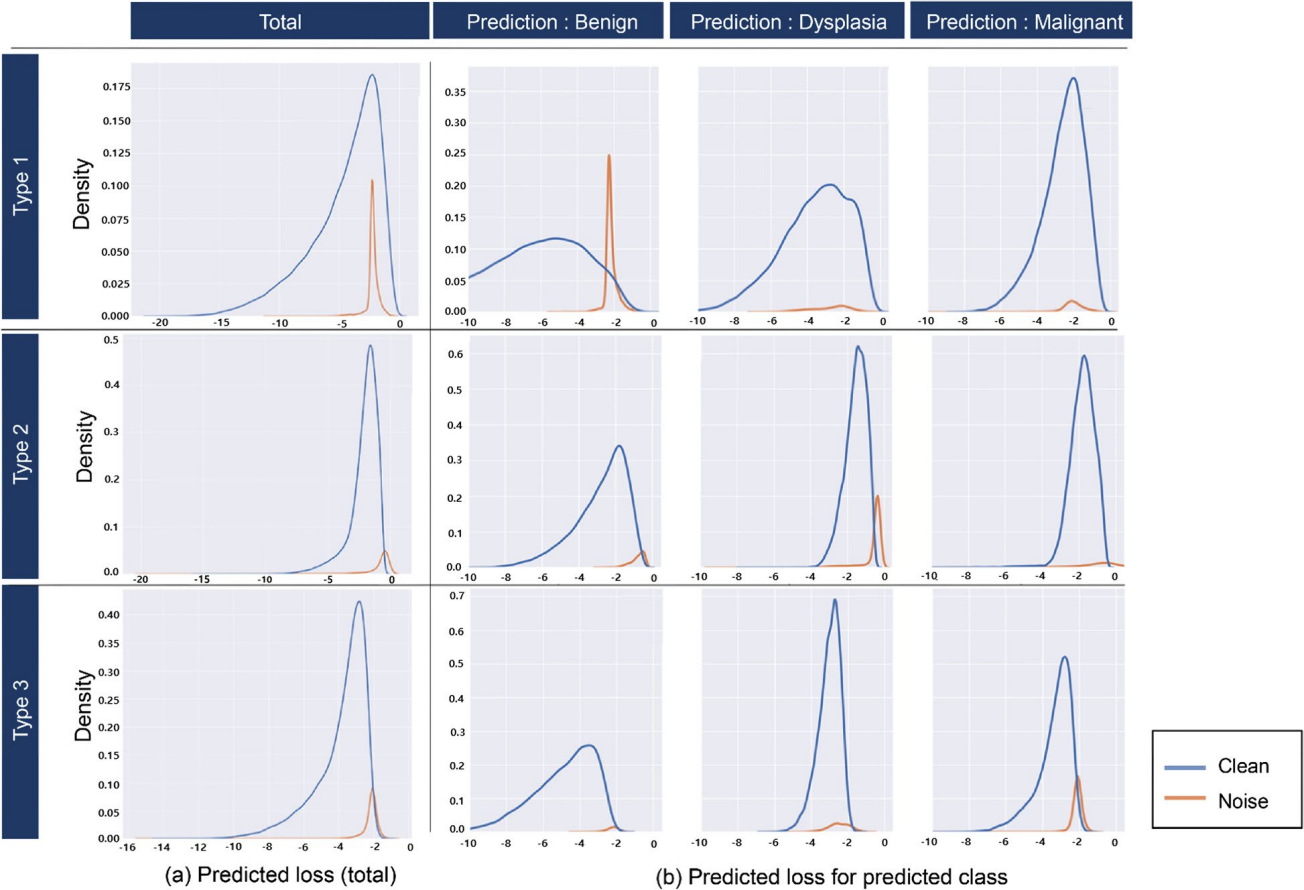
Example of the distribution of predicted loss values (**a**) Distribution types when predicting the loss with a trained LPM on the entire unlabeled dataset. Most of the noisy data aligns with the high-loss location similar to informative data. **b** Distribution obtained by predicting the loss with the same trained LPM as in (**a**) and classifying it into predicted classes. Within these predicted classes, it can be observed that noisy data are distributed in the higher loss location compared to informative data

We first compared the predicted loss values of trackable noisy data and clean data included in ${D}_{U}$. Representative types of distributions were chosen as typical examples from the distributions generated during three trials of the AL method (LL) with 10 iterations each, resulting in a total of 30 iterations for ${D}_{U}$ (Fig. 6). Most noisy data were found to have relatively high predicted loss values. Figure 6(a) shows the result of operating the LPM for the entire unlabeled data, while Fig. 6(b) shows the result of classifying the same data by the predicted class. We identified three main types of noisy data, with Type 1 showing a large distribution of noisy data in the middle position. When classifying them by predicted class and creating a distribution (Fig. 6[b]), each class still had a large number of distributions at a high-loss location. Therefore, we expected that different thresholds would work for each class, as we proposed. Thus, the proposed method included the selection of clean and beneficial data below the threshold by computing different thresholds for each class.

### Sensitivity analysis for alpha

Tables 2 and 3 present the results of a sensitivity analysis based on alpha values, along with the corresponding noise selection results. Alpha represents the weight value ${a}_{t}$ that determines the threshold in Eq. (5). We evaluated the model's performance and the ratio of noise selection by incrementally increasing this weight value from a low starting point. In the ‘no threshold’ case, the model had a high proportion (35%) of noisy data. Table 3 shows the average number of noise selections that occurred in 10 iterations and five trials. For instance, the value “353.1” means that, on average, 353 out of 1,000 noisy samples were selected. As a result, the average performance was low, and the model’s performance was relatively unstable. When we increased the alpha value, we observed that the rate of noise selection decreased. However, performance no longer improved in larger alpha intervals (***a*** = 1.1 and 1.2). As the alpha value increased, the model tended to select data with relatively low loss values, which reduced the benefits of the selected data and failed to effectively increase performance. Nevertheless, the proposed method achieved high performance with a low noise selection ratio. We applied different alpha values for each class, using “1.0” for Benign, “1.1” for Dysplasia, and “1.0” for Malignant.

**Table 2:** Sensitivity analysis for alpha (accuracy)

Noisy dataset								Clean dataset	
*a*	No threshold (LL)	0.5	0.8	1	Proposed	1.1	1.2	LL	Proposed
1	0.810 ± 0.012	0.812 ± 0.010	0.808 ± 0.014	0.815 ± 0.015	0.803 ± 0.007	0.811 ± 0.009	0.808 ± 0.017	0.805 ± 0.004	0.809 ± 0.004
2	0.794 ± 0.040	0.861 ± 0.006	0.817 ± 0.028	0.850 ± 0.020	0.856 ± 0.027	0.833 ± 0.025	0.839 ± 0.023	0.786 ± 0.124	0.846 ± 0.010
3	0.821 ± 0.035	0.808 ± 0.137	0.847 ± 0.030	0.868 ± 0.014	0.864 ± 0.032	0.842 ± 0.034	0.863 ± 0.015	0.855 ± 0.042	0.873 ± 0.013
4	0.815 ± 0.064	0.866 ± 0.011	0.876 ± 0.020	0.880 ± 0.014	0.881 ± 0.026	0.863 ± 0.017	0.860 ± 0.019	0.897 ± 0.012	0.892 ± 0.011
5	0.857 ± 0.044	0.675 ± 0.222	0.892 ± 0.010	0.881 ± 0.013	0.893 ± 0.018	0.892 ± 0.005	0.876 ± 0.029	0.827 ± 0.098	0.902 ± 0.019
6	0.859 ± 0.018	0.898 ± 0.010	0.879 ± 0.015	0.885 ± 0.028	0.899 ± 0.009	0.897 ± 0.009	0.887 ± 0.012	0.894 ± 0.027	0.892 ± 0.031
7	0.843 ± 0.048	0.870 ± 0.027	0.879 ± 0.035	0.905 ± 0.005	0.897 ± 0.021	0.893 ± 0.014	0.888 ± 0.011	0.903 ± 0.007	0.910 ± 0.009
8	0.897 ± 0.019	0.894 ± 0.025	0.906 ± 0.004	0.901 ± 0.014	0.908 ± 0.007	0.892 ± 0.022	0.873 ± 0.020	0.907 ± 0.010	0.912 ± 0.015
9	0.882 ± 0.045	0.882 ± 0.033	0.894 ± 0.021	0.904 ± 0.015	0.914 ± 0.007	0.878 ± 0.045	0.895 ± 0.010	0.882 ± 0.033	0.908 ± 0.019
10	0.863 ± 0.062	0.890 ± 0.019	0.898 ± 0.014	0.917 ± 0.006	0.917 ± 0.004	0.899 ± 0.016	0.888 ± 0.008	0.896 ± 0.014	0.924 ± 0.005

Table 2 shows the change in the accuracy of the model according to the change in the alpha value. We obtained the mean and standard deviation at each iteration after five trials for one alpha value. One trial includes 10 iterations and after 10 iterations, the next trial can be processed

*LL* Learning loss

**Table 3:** Sensitivity analysis for alpha (noise selection)

	0 (LL)	0.5	0.8	1	Proposed	1.1	1.2
Mean	353.1	116.4	96.3	80	29.3	24.9	16.6
Total	3177.6	1047.4	866.6	720.4	263.4	224	149.6
N-Ratio	35.31%	11.64%	9.63	8.00%	2.93%	2.49%	1.66%

Table 3 shows the number of noise selections by the model in five trials. The “Total” item is the average of the total number of noise selections in the five trials. In each trial, the first iteration is excluded from the noise selection amount analysis because clean data are used fixedly at the first iteration. Therefore, the “mean” item is a value obtained by dividing the total item value by nine. The N-ratio refers to the percentage of noise selected, in other words, the ratio of noisy data out of the 1,000 data points selected by the model in each iteration.

### Comparative performances of the AL methods

**Fig. 7:**
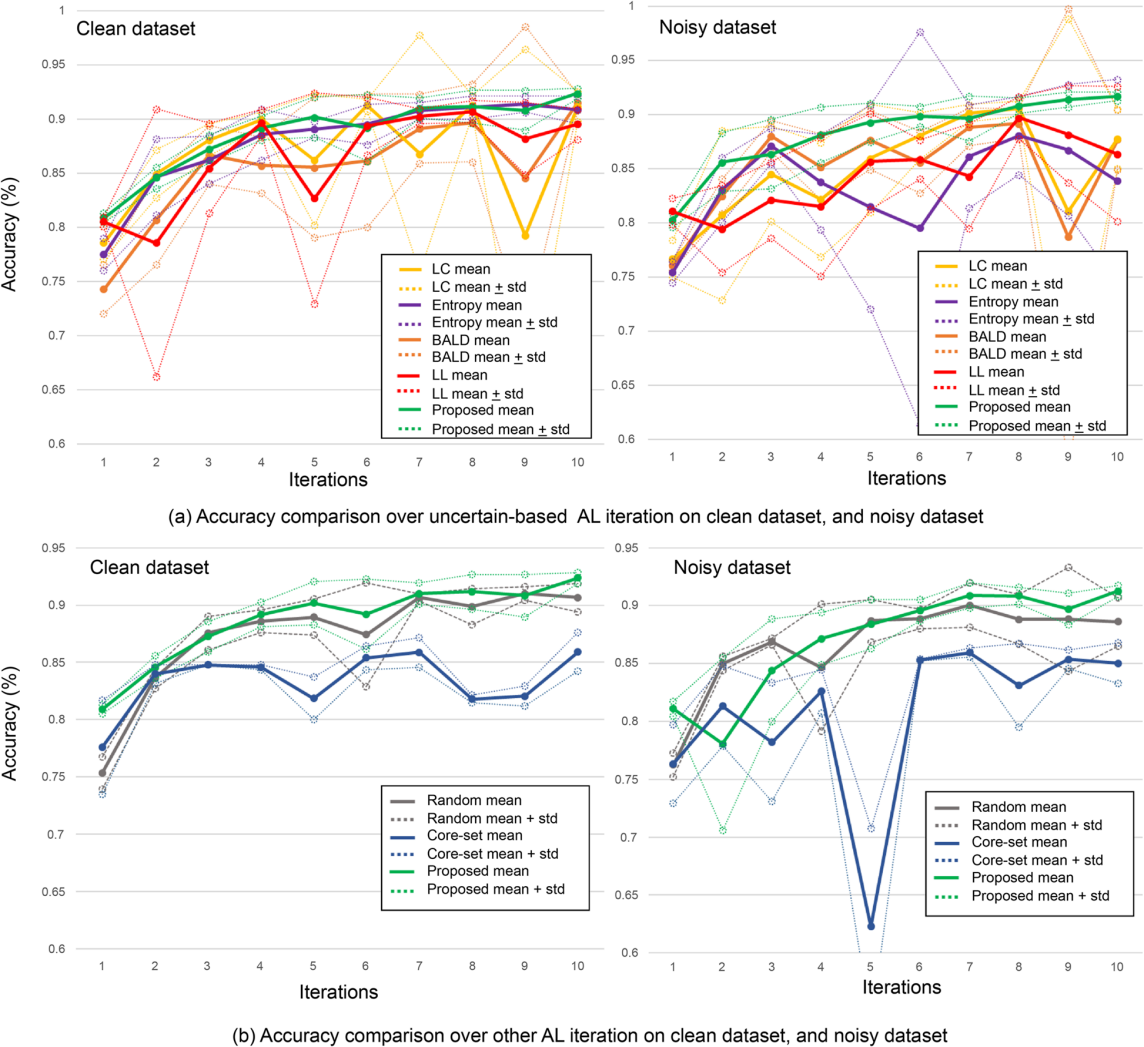
Accuracy comparison over AL methods with clean and noisy datasets. **a** Comparison between the proposed and uncertainty-based methods. The results on a clean dataset are presented on the left, while the results on a noisy dataset are displayed on the right. In each experimental trial, there were 10 sampling iterations, and this process was repeated five times for each method to calculate the accuracy. The figure shows the average and standard deviation of each iteration from the five trials. **b** Comparison of random sampling, coreset, and proposed methods, using the same experimental setup as in (**a**). *Abbreviations AL* active learning, *LC* least confidence*, BALD*, Bayesian active learning by disagreement, LL: learning loss; std: standard deviation

The performances of five alternative AL methods (i.e., LC, entropy, BALD, core-set, random, and proposed) were first evaluated by analyzing the mean and standard deviation of the accuracy obtained from each of the models. We compared the accuracies of the methods in clean and noisy datasets, and also compared and analyzed the amount of noisy data among the data selected by each of the AL methods. Figure 7 presents the accuracy measurements of each AL method in an environment where real-world noise can be used. We measured the performances at each iteration while increasing the size of the labeled data over 10 iterations. In the first iteration, we trained all models with the same clean data. Additionally, we conducted experiments five times for each method and calculated their means and variances. More specifically, Fig. 7(a) compares the uncertainty-based AL method (LC, entropy, BALD) with the proposed method. These AL methods operating on clean datasets had a performance of over 90% in the 10th iteration, which was slightly lower than that of the proposed method. However, when we conducted the same experiment on the noisy dataset, the overall average performances of these AL methods decreased, and the variance increased. In contrast, the proposed method confirmed that the difference in tendency between the results of performing AL with clean data and the results of performing AL with noisy data was not noticeable and the variance remained low. Figure 7(b) compares the core-set and RS methods, which are representativeness-based methods, with the proposed method. The core-set method had a low overall performance due to the large amount of computation required. In the case of the RS method, it showed a higher variance when learning from a clean dataset and a decrease in stability when learning from a noisy dataset. Both methods showed poor performances, relative to the proposed method.

Figure 7 (b) shows that both the representativeness-based method and the RS method, which induced the selection of various data, showed poor overall performances, supporting the idea that selecting data with higher uncertainty than representativeness is more effective in improving the model’s performance in learning pathological tissue image classification. However, a relatively low variance was observed in the core-set and RS methods, indicating that learning various data with representations of the stability of model performance changes had a lasting effect on model training.

The entropy and the proposed methods had an accuracy of over 90% from the seventh iteration on the clean dataset and maintained a performance of more than 90% until the 10th iteration, the final iteration. Moreover, the proposed method showed a steady increase in performance up to the 10th iteration, where it had its highest performance. For some AL methods, the performance did not increase gradually and, in general, we observed a higher variance than with the proposed method, implying that those models’ performances can be highly sensitive to the data selected in each iteration.

Moreover, Fig. 7 provides a comparison of the performance changes on the noisy dataset. Most AL models showed a decrease in average performance and an increase in variance. In contrast, the proposed method demonstrated robust performance against real-world noise, with only a slight performance reduction on the noisy dataset. Specifically, the proposed method had 91.7% accuracy on the noisy dataset, revealing a performance reduction of only 0.7% compared to when it was trained under the clean dataset condition. In contrast, other AL methods had a 2% to 7% performance reduction on the noisy dataset when compared to the clean dataset condition.

### Comparative noise selection of the AL methods

**Fig. 8:**
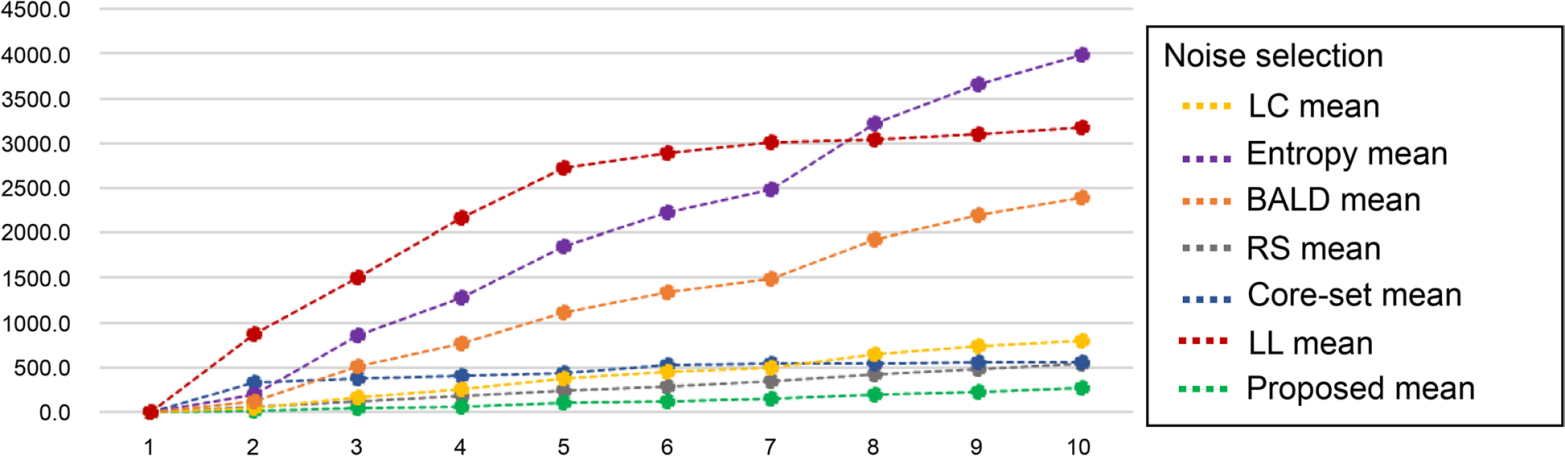
A cumulative graph depicting the number of noise data selections over iterations by alternative AL methods. AL: active learning; LC: least confidence; BALD: Bayesian active learning by disagreement; RS: random sampling; LL: learning loss

To delve into the performance differences under the noisy conditions, we recorded the amount of noisy data selected by each AL method during the aforementioned experimental process and compared the results. The results are graphically summarized in Fig. 8.

Further, Fig. 8 shows the cumulative quantity of noisy data selected by each AL method in terms of iterative performance. Methods that measure uncertainty, such as BALD and entropy, consistently selected a significant amount of noise compared to other methodologies. The core-set and RS methods tended to select a relatively small amount of noisy data because various data were selected. Nevertheless, the proposed method, an AL method for selecting data, tended to select the smallest amount of data, except for intervals where noisy data were prevalent. Table 3 shows the average amount of noisy data selected in each iteration. For the first iteration, the same 1,000 clean data points were used, thus it was not included in the average calculation. Additionally, the ratio of noise selected in each iteration was calculated and is presented in the N-Ratio row of Table 3. N-Ratio represents the ratio of the number of data corresponding to noisy data among the 1,000 data points selected for each iteration and was calculated using the following equation.

10 $$N-Ratio= \frac{(mean\, of \,noise \,slection)}{1000}\times 100$$

As shown in Table 4, the proposed method selected an average of 29.3 noisy data points out of 1,000 data points in each iteration, resulting in an average noisy data ratio of 2.93% and a clean data selection ratio of 97.07%. In contrast, the entropy method selected approximately 4,000 noisy data points throughout the experiment, which means that more than 400 noisy data points were selected per iteration. Therefore, the labeled data obtained by the entropy method only accounted for approximately 56% of the total data, which was significantly lower than the proposed method’s labeling acquisition rate of approximately 97%. Additionally, in the case of the core-set or RS method, various data were selected, resulting in a noisy data ratio of approximately 6%, which was similar to the 5.8% ratio of the total data.

**Table 4:** Number of noisy data selections by the AL methods in each iteration

Iteration	LC	Entropy	BALD	LL	RS	Core-set	Proposed
1	0.0	0.0	0.0	0.0	0.0	0.0	0.0
2	38.2	191.0	114.6	878.4	60.3	326.3	15.2
3	132.0	660.0	396.0	626.4	57.8	55.0	28.2
4	86.1	430.6	258.3	667.8	61.8	17.7	20.4
5	114.3	571.4	342.8	558.4	57.5	30.3	36.0
6	74.6	373.0	223.8	161.2	53.3	101.3	25.8
7	50.7	253.4	152.0	115.2	60.0	9.3	31.0
8	148.2	740.8	444.5	35	65.5	7.7	33.8
9	88.5	442.6	265.6	62	60.5	7.7	37.0
10	67.0	335.2	201.1	73.2	59.0	6.0	36.0
Mean	88.8	444.2	266.5	353.1	59.5	62.36	29.3
Total	799.6	3998	2398.8	3177.6	535.5	561.3	263.4
N-Ratio	8.9%	44.4%	26.7%	35.3%	6.0%	6.2%	2.93%

*LC* Least confidence, *BALD* Bayesian active learning by disagreement, *LL* Learning loss, *RS* Random sampling, *N-Ratio* Noise-ratio

### Comparisons of performance in the final iteration

Table 5 presents the accuracy scores of all of the aforementioned methods obtained in the final iteration for clean and noisy datasets. The methods that measure uncertainty, such as BALD and entropy, consistently selected a significant amount of noise compared to the other methods. The core-set and RS methods tended to select a relatively small amount of noisy data because a large number of noisy data was distributed in sections of relatively high uncertainty values and various levels of data were selected by the two methods. Nevertheless, the proposed method tended to select the smallest amount of noisy data, except for intervals where noisy data were prevalent. Table 6 also shows the number and proportion of noisy data selected by each method. A high N-ratio means that more noisy data is included in the selected data. The proposed method showed the lowest level of noise selection among the alternative AL methods.

**Table 5:** Accuracy and standard deviation scores obtained in the final iteration for clean and noisy datasets

Dataset	LC	Entropy	BALD	RS	Core-set	LL	Proposed
Clean	0.912 ± 0.014	0.909 ± 0.013	0.916 ± 0.007	0.907 ± 0.012	0.859 ± 0.017	0.896 ± 0.014	0.924 ± 0.005
Noisy	0.878 ± 0.027	0.877 ± 0.028	0.838 ± 0.094	0.886 ± 0.021	0.850 ± 0.018	0.863 ± 0.062	0.917 ± 0.004

*Abbreviations LC* Least confidence, *BALD* Bayesian active learning by disagreement, *RS* Random sampling

**Table 6:** Total noise selection by each AL method

	LC	Entropy	BALD	RS	Core-set	LL	Proposed
Mean	88.8	444.2	266.5	59.5	62.36	353.1	29.3
Total	799.6	3998	2398.8	535.5	561.3	3177.6	263.4
N-Ratio	8.90%	44.40%	26.70%	6.00%	6.20%	35.3%	2.93%

*Abbreviations AL* Active learning, *LC* Least confidence, *BALD* Bayesian active learning by disagreement, *RS* Random sampling

## Discussion

The objective of this study was to develop a robust AL method against a noisy histopathological dataset. Constructing a CNN-based DL system requires a pathologist to perform image-labeling tasks [15, 20, 27]. AL has been studied to reduce the workload of the labeling by selectively labeling data that are more effective for learning, which lowers labeling costs and reduces the workload of an oracle [7, 22, 32]. However, in real-world industrial environments, various forms of noisy data are included in WSIs [24, 25, 33]. If the model selects noisy data, an oracle’s workload is not effectively reduced. To address this problem, this paper proposes a new method to select data with predictive losses below a certain threshold to develop a robust AL method.

Pathological datasets can be noisy for a variety of reasons, such as interference during image capture or data conversion, mislabeled data, or out-of-distribution samples. Previous studies have addressed this issue by removing noise from the images or separating the noisy data. There are studies aimed at alleviating the interfering image problems by utilizing generative model-based methods and removing perturbations that occur within the image [34–37]. On the other hand, there are studies that have also been conducted to separate noisy data. These studies utilize the features of noisy data in a dataset to perform different processing on noisy vs. clean data and to filter out noisy data from clean data [38, 39]. Additionally, Ponzio et al. [33] applied Bayesian neural networks to measure the uncertainty of the data and to remove data with high uncertainty. Through this method, they filtered spurious data, such as blood, fat, glass, and stroma, for pathological classification. Ashraf et al. [27] proposed a patch-data cleaning method called LossDiff, which automatically sets an appropriate threshold based on the batch average loss for each class.

The proposed AL framework combined uncertainty-based AL with uncertainty-based data-filtering methods. We adopted LL [26] as an informative image selection method, which has several advantages. First, it is simple and task-agnostic, making it suitable for use with deep networks. Deep networks are trained by minimizing a single loss, regardless of task type, number of tasks, or complexity of architecture, which makes LL useful for various purposes, as long as the LPM can be attached. Secondly, LL predicts loss values for unlabeled data, making it possible to calculate thresholds for filtering data collected during the training stage. Finally, LL can be utilized after training to select informative data.

We also focused on data-filtering methods among various existing data-cleaning methods. We used a modified version of LossDiff [27]. We induced the model to generate thresholds by collecting the predicted loss in the training stage and selecting data with lower-than-threshold loss values in the sampling phase, thus allowing the model to select appropriate and informative training data. This is important for robust AL on real-world noisy datasets where noise and informative data are mixed. Most data filtering methods consider data with high uncertainty to be noisy data, which have a high potential for both noisy and informative data. Therefore, our proposed framework ensured that the model selected informative data and filtered out noisy data.

Additionally, we confirmed the tendency of the predicted loss values of the noisy data and clean data and their distribution. The results showed that the noisy data were distributed in a high uncertainty (i.e., high predicted-loss) section in each prediction class distribution. Therefore, different threshold settings were required depending on the prediction class for proper threshold generation. Subsequently, a sensitivity analysis was performed on the alpha value to select an appropriate value. The results of the sensitivity analysis demonstrated a performance change in the proportion of noisy data in the selected data as the alpha value gradually increased. We experimentally found alpha values that showed good performance, while also selecting less noise, and showed the best results when the values were set differently, according to the predicted class (Benign: “1.0”, Dysplasia: “1.1”, Malignant: “1.0”).

In the 10th iteration on the noisy dataset, our model had an accuracy of 0.917, with a performance reduction of less than 1% (0.924–0.917), and it was confirmed that only 29.3 noisy data points were selected on average from 1,000 selected data points. We performed comparisons with other methodologies in repeated experiments with corresponding alpha values. The proposed method showed indistinguishable performance differences on clean datasets and noisy datasets, and a lower numerical noise selection ratio than other AL methods. The study results clearly showed that the performance of the model generated by the proposed method was robust, even in noisy environments.

Tables 5 and 6 provide an overview of the results from our experimental study. On the 10th iteration on the noisy dataset, the proposed method showed a mean of accuracy and standard deviation of 0.917 and 0.004, respectively, unlike other methods with a 3–7% performance reduction from the clean dataset. Our proposed method exhibited only a slight performance reduction of 0.7%. These findings support the notion that our proposed method is more robust than existing methods with noise. For the core-set method, the 10th iteration had an accuracy of 0.850, which was approximately 6% lower than the performance of the proposed model. Moreover, for the uncertainty-based methods, the differences in performance between the noisy and the clean datasets were larger. In the case of BALD, the most affected by the noise, a performance reduction of approximately 7% was found. The proposed method demonstrated little variation in performance on both clean and noisy datasets, with a lower numerical noise selection ratio than the other AL methods. In contrast, the proposed method achieved the highest accuracy on both noisy and clean datasets, while maintaining the smallest performance decline against the noisy dataset. Furthermore, we observed that our proposed AL method selected the least amount of noisy data.

Notably, although LC and entropy, which are uncertainty-based AL methods, differ significantly in their noise selection ratios, the differences in performance were negligible. Furthermore, even with BALD, which selected even fewer noisy data points than entropy, the results showed that selecting less noise was not a guarantee of good performance. This point was further emphasized by the core-set and RS methods. By performing two functions simultaneously, beneficial data selection and noisy data avoidance, and in a straightforward manner, our proposed method yielded high performance and selected more labeled data. In fact, given that only 56% of the labeled data was obtained by the extant entropy method, the actual amount of data acquired was only approximately half of that by the proposed method. In contrast to the average of 3,998 noisy data points selected out of 10,000 by the entropy methods, it was confirmed that only an average of 263.4 were selected by the proposed method, indicating that utilizing existing AL methods on real-world noisy datasets would increase the workload of the oracle due to the need for more labeling tasks. At the same time, the proposed method minimized the increase in the oracle’s workload by performing AL in noisy environments, with an average clean data selection rate of 97.07%.

The proposed method can be applied to training an image classification model aptly in real-world industrial practice with noisy data. We experimented with alternative AL strategies using patch images generated from real-world WSIs, enabling us to test the proposed method against real-world noisy data and understand its performances and general tendencies, relative to the state-of-the-art methods. Among them, the proposed method showed better performances in terms of noise selection levels and accuracies.

Nevertheless, this study’s proposed method has certain limitations and boundary conditions that need to be noted. We used predictive losses collected during the training process to create thresholds and exclude data above them from candidate data, to avoid selecting noisy data. However, there can be noises that are difficult to classify, thereby making it difficult to achieve good model performance. For example, in the case of noise labels that arise during the oracle’s labeling process, the quality of the labeled data degraded pose an inherent risk to the collected label data. To reduce noise labels that may occur in various AL scenarios, the data cleaning method needs to be extended to the AL training data. Additionally, the proposed method focused on quantitatively reducing the workload of the oracle. However, considering that the task weight caused by the actual labeling process is not only affected by the amount of data, it is also necessary to consider how to qualitatively reduce the task weight during the labeling process. Therefore, in our future studies, we plan to analyze the differences between the data that experts find beneficial and those that the AL method judges to be beneficial.

## Conclusions

In this study, we propose a novel AL method for pathological image classification that minimizes noisy data selection when querying data from an unlabeled set. Our model selected data with high informativeness while avoiding the distribution interval of noisy data, by taking the characteristics of the predicted loss values of the noisy patches occurring in the field into account. When we trained our model using this method, it achieved an accuracy of 91.7% on the noisy dataset and 92.4% on the clean dataset in the final iteration, resulting in a performance reduction of less than 1%.

With its reduced noise selection ratio and increased accuracy, the proposed method may contribute to relieving the workload of pathologists in the context of AL applied to automated image processing for cancer detection in the workplace. The data reflected the actual level of noise embedded in the WSIs created for a large medical diagnosis organization in Korea. Against this dataset collected from a real workplace, the proposed method produced a superior and more robust performance, compared to the state-of-the-art methods. The study findings are expected to be applicable to other pathological image processing areas, even though the proposed method was tested against stomach images generated by a high-quality medical scanner. Furthermore, the DL model training system proposed in this study has the potential to enhance the working environment for pathologists while continuously improving DL models. This advancement can result in better resource utilization, increased productivity, and ultimately benefit both pathologists and the patients they care for.

## Data Availability

Patient clinical data and whole slide images that support the findings of this study cannot be made publicly available due to ethical concerns regarding patient privacy. The datasets collected and analyzed during the current study are not publicly available due to limited computing, storage resources, and the security policy of Seegene Medical Foundation. However, they are restrictively available from the Seegene Medical Foundation Institutional Data Access/Ethics Committee (contact via corresponding authors) for researchers who meet the criteria for access to confidential data.

## Abbreviations

AL: Active learning
BALD: Bayesian active learning by disagreement
CNNs: Convolutional Neural Networks
DL: Deep learning
LC: Least confidence
LL: Learning loss
LPM: Loss prediction module
PLCM: Predicted loss collection module
RS: Random sampling
s/f: Suspicious for
s/o: Suspicious of
WSIs: Whole-slide images
